# Mesenteric cyst presenting with acute abdomen pain and bowel obstruction: Case report and brief literature review

**DOI:** 10.1016/j.amsu.2020.09.001

**Published:** 2020-09-06

**Authors:** Nubyhélia Maria Negreiro de Carvalho, Jose Airton Lopes Filho, Isabella Cabral Marinho Plens, Victor Ary Camara, Cayo Cesar de Gois Teixeira, Paulo Henrique Dourado Figueiredo, Olavo Napoleão de Araujo Junior

**Affiliations:** a Department of Digestive Surgery, Hospital Geral de Fortaleza, Brazil; b School of Medicine, Universidade Federal do Ceara, Brazil; c School of Medicine, Universidade de Fortaleza, Brazil; d Preceptor of the Department of Digestive Surgery, Hospital Geral de Fortaleza, Brazil; e Chief of the Department of Digestive Surgery, Hospital Geral de Fortaleza, Brazil

**Keywords:** Mesenteric cyst, Abdominal mass, Mesothelioma, Lymphangioma

## Abstract

Mesenteric cysts were first described in 1507 and since then remain as a rare intraabdominal pathology. The etiology of this kind of tumor is still unclear and the classification remains controversial. They are usually asymptomatic, but can also cause acute abdominal pain and sometimes need emergency surgical approach. Clinical history, physical exam and complementary tests do not always provide diagnosis, which in many cases is made after surgery. Surgical management with complete excision of the cyst is the gold standard treatment. Laparoscopy technique should be preferred whenever is possible. The knowledge of these rare tumors is important for considering the correct approach. The goals of this article is to describe a case report of mesenteric cyst presented with acute abdominal pain at the emergency and do a brief literature review about this entity.

## Highlights

• Mesenteric cysts are a rare cause of intraabdominal mass.
• Mesenteric cyst should always be considered it whenever the patient has an intraabdominal palpable mass.
• Computed tomography of the abdomen can help find the diagnosis in many cases.
• Even when mesenteric cyst is diagnosed incidentally, surgery should always be indicated in order to prevent complications.
• Surgery is the treatment of choice and recurrence rate is low when the correct treatment is performed.

## 1 Introduction

Mesenteric cyst is a rare pathology that is asymptomatic in most of the cases and affects any segment of the bowel mesentery, but it is more prevalent in the ileum [[1], [2], [3], [4]]. It was first described by Benevieni in 1507 [1,[4], [5], [6], [7]]. Tillaux described the first successful surgery for a mesenteric cyst in 1880 [1,[5], [6], [7], [8]] and finally the first marsupialization of a mesenteric neoplasm was performed by Pean in 1883 [5,6]. The mesenteric cysts can occur at any age [7,9]. and its incidence reaches around 1/100.000 in adults [[1], [2], [3],^6^]. There seems to be no difference in involvement between sexes [1,8]. The cyst has benign behavior in almost all cases and it pathogenesis is still unknown [1,2,4,6]. They are often asymptomatic and found incidentally when patients are undergoing investigations or treatment of another condition [7], but it can complicate and start manifesting different symptoms, including an acute abdomen [1,7,10,11]. The treatment of choice is complete surgical removal of the lesion, which also offers histopathological confirmation of the diagnosis [1,7,11]. Thus, this article purpose is to report a case of a patient that presented with acute abdomen at the emergency caused by a bulky mesenteric cyst. The work has been reported in line with the SCARE criteria [12].

## 2 Case report

A 33-year-old male patient with no significant past medical history presented to the Emergency Department due to severe abdominal pain during the last five days. The pain was located in the left flank, radiated to the back, associated with difficult evacuation and one episode of bloody vomiting. He denied weight loss, fever and other constitutional symptoms.

On physical examination a large abdominal mass could be felt occupying from the epigastrium to the hypogastrium, in addition to both flanks, being slightly lateralized to the left, with well-defined margins, fibroelastic consistency, besides being mobile and slightly tense and painful on palpation, probably due to its great extension. There were no signs of peritonitis.

Blood tests including full blood count serum electrolytes, canalicular enzymes, bilirubin, pancreatic enzymes and liver and kidney function tests were performed which, besides a hemoglobin level of 10.5g/dL (reference range: 14–18 g/dL), were unremarkable and showed no other abnormalities. Prothrombin time, partial thromboplastine time and INR (International Normalized Ratio) were slightly increased. As for tumor markers, Carcinoembryonic Antigen (CEA) level was 0,52ng/mL (reference range: <3,5ng/dL) and Carbohydrate Antigen 19-9 (CA 19-9) was 3,92U/mL (reference range: <35U/mL).

**Fig. 1:**
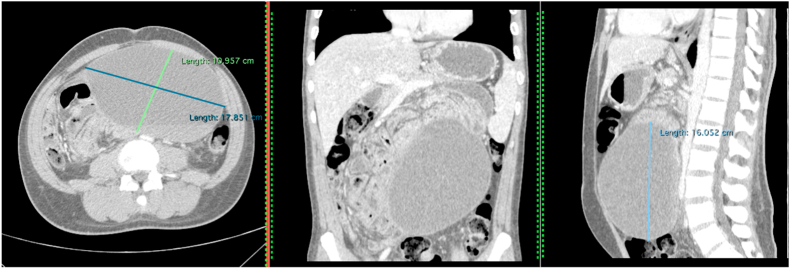
Abdomen Contrast-CT demonstrating a well-defined large cystic lesion causing compression of intestinal loops with close relation with the Treitz ligament and middle colic vessels, measuring 17, 8 × 16 × 10, 9 cm.

During the investigation, a computed tomography (CT) was requested, which exhibited a large predominant cystic lesion, with solid component in posterior region, measuring 17, 8 × 16 × 10, 9cm, located mainly in the mesogastrium causing compression of intestinal loops, suggesting the diagnosis of mesenteric cyst (Fig. 1).

**Fig. 2:**
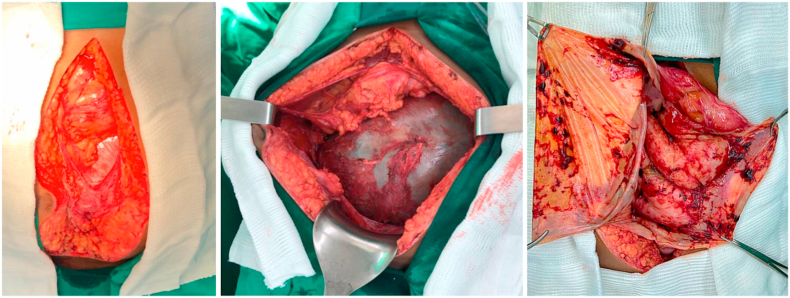
Intraoperative images of mesentery cyst in 3 moments: (a) after the skin section with peritoneum adhesions; (b) showing well-defined wall after superior and lateral dissection; (c) aspect of the posterior wall of the cyst connected with the root of the mesentery after drainage of the cyst fluid.

The resection of the cystic lesion was indicated. Laparoscopic approach was not attempted due to the size of the lesion. At surgical approach through median incision, a large cyst with peritoneum and small bowel adhesions was found. After anterior and lateral dissection and complete liberation of the colon, the anterior wall of the cyst was incised and 1.500 mL of a dark brown fluid was drained out and sent for further analysis. The posterior wall of the cyst was firmly connected to the root of the mesentery, adjacent to the middle colic vessels, making it impossible to safely resect it and was opted to perform cystic marsupialization (Fig. 2).

The total operative time was 125 minutes and the patient was discharged home three days after the procedure without intercurrences.

**Fig. 3:**
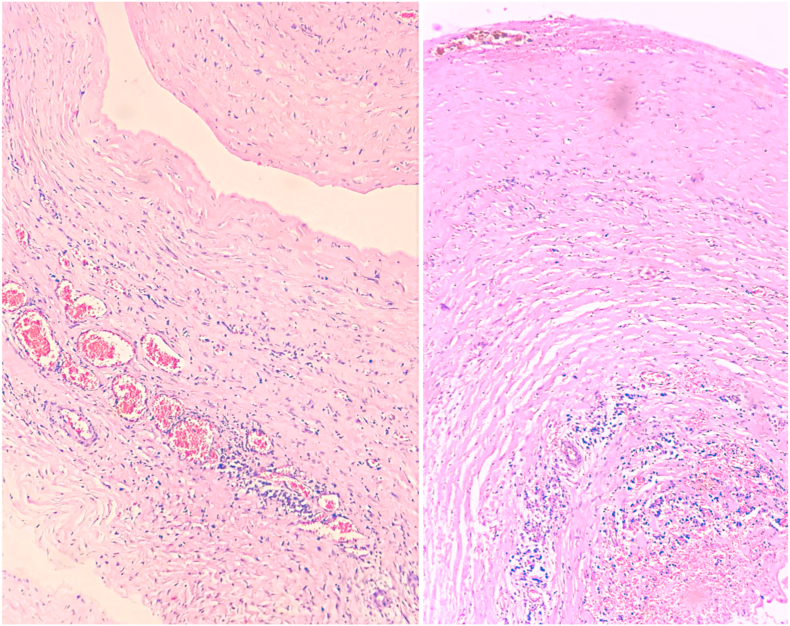
Cyst wall constituted by dense fibrous connective tissue, containing (a) lymphomononuclear inflammatory infiltrate permeating congested thin-walled blood vessels and (b) hemorrhagic foci and accumulations of hemosiderophages (Hematoxylin and eosin; 10 × 10).

In the analysis of the cyst fluid, hypocellular cytological preparations exhibited rare lymphocytes and histiocytes, on a sero-hemorrhagic background. No epithelial elements or cytological signs of malignancy were identified in the material examined. Histopathological examination of cyst capsule revealed a dense fibrous connective wall with indistinct epithelial lining, besides of frequent thin-walled congested vessels, lymphocytic inflammatory process with associated hemosiderophages and no signs of malignancy (Fig. 3).

The findings were consistent with the diagnosis of benign cystic mesothelioma of the mesentery. At a post-operative follow up period 3 months, there were no significant symptoms and no clinical evidence of recurrence.

## 3 Discussion

Although the first description of mesenteric cyst was in the 16th century [5,9], its origin is still unclear [1,4,9,13]. Some authors postulate that they are a consequence of a continual growth of congenitally malformed or malpositioned lymphatic tissue, while others believe that they can originate secondary to trauma, degenerating lymph nodes or failure of the leaves of the mesentery to fuse properly [1,4,9].

Mesenteric cysts can be located anywhere in the mesentery from the duodenum to the rectum [[1], [2], [3], [4],^7^,^9^], and it may extend from the base of the mesentery into the retroperitoneum [[3], [4], [5]], but it is usually located in the small bowel mesentery [1,4,5,8,9].

The incidence of malignant cysts is low (less than 3% of cases) [[1], [2], [3], [4], [5],^9^,^13^] and more common in adults [5]. They are usually sarcomata since the cellular elements of the mesodermal origin, but rare cases of adenocarcinoma have been described [9].

Many authors consider mesenteric, omental, and retroperitoneal cysts as one group because they derive from the same embryological structures, while others believe that the retroperitoneal lesions represent another group of pathology [6].

Several classifications have been reported. The most widely recognized divides mesenteric cysts into four groups based on clinical and etiological features: (1) embryonic and developmental; (2) traumatic; (3) neoplastic, and (4) infectious [1,6].

They can also be differentiated into lymphatic cysts and lymphangiomas or simple mesothelial cysts and benign cysts. Simple lymphatic and mesothelial cysts usually remain stable and asymptomatic over time, as lymphangiomas and benign cystic mesotheliomas may have aggressive and invasive properties. Histopathologically, flat endothelial cells characteristically line cysts of lymphatic origin with a wall containing smooth muscle fibers, lymphoid tissue, and/or occasional foam cells. In contrast, flat, cuboidal, or columnar cells line cysts of mesothelial origin and their wall is fibrous without any lymphatic structures. The distinction between simple lymphatic cysts and lymphangiomas or between simple mesothelial cysts and benign cystic mesotheliomas is based on macroscopic characteristics. Simple cysts are usually small (1–5 cm) and unilocular, whereas lymphangiomas and benign cystic mesotheliomas are large and include multiple cysts of several sizes. Occasionally, their gross appearance may be similar and the distinction is not possible [6].

Mesenteric cysts can be single or multiple and uni or multi-loculated and the contents of the cyst vary from clear to milky or dark brown depending on the location of the cyst and the presence or absence of hemorrhage [9].

Although often asymptomatic, 10% of the patients present as an acute abdomen [3]. They can also appear as non-specific abdominal features [[1], [2], [3],^11^], and as an incidental finding of an abdominal mass [9,13].

The size of the cysts and age of the patient influence the clinical presentation [6,9]. Abdominal pain, abdominal distention, fever, nausea, constipation, diarrhea are some of the symptoms that this pathology can cause [[5], [6], [7], [8],^13^].

Children usually present with acute abdomen, while adults have more indolent symptoms [6]. The cyst complications that can lead to an acute abdominal presentation can be spontaneous rupture, infection, hemorrhage, cyst torsion, herniation of the cyst, intestinal ischemia and abdominal obstruction by compression of the lesion [1,7,8,10]. In more than 50% of the cases an abdominal mass can be palpable on the physical examination [1,6,9].

The clinical history and physical exam may suggest the diagnosis, but in many cases the diagnosis is made by the exclusion of other causes or only after surgery [5]. The diagnosis can be challenging due to rarity, lack of specific symptoms and variability in location and size [4,11].

Laboratory tests contribute little to the diagnosis [2]. Ultrasonic imaging and computed tomography can help find the correct diagnosis of mesenteric cyst [1,2,4,5,[7], [8], [9], [10]], although there is no specific radiologic signs [4,9]. If bleeding has occurred, debris can be seen in the cyst fluid [1].

The surgical treatment is the complete excision of the mesenteric tumor [[1], [2], [3], [4], [5], [6], [7], [8], [9], [10], [11],^13^] either laparoscopically or through a laparotomy [7]. The type of the surgery depends on the size of the cyst, its location in the peritoneal cavity and the surgeon experience [13]. In some cases a simultaneous intestinal resection it is necessary for the excision to be complete [[3], [4], [5],^9^,^10^,^13^]. Even in malignant lesions, the enucleation is sufficient in most cases and is associated with a high cure rate [9]. When none of those options is possible, marsupialization of the lesion is accepted as a treatment option [5,9].

Partial excision is associated with recurrence and morbidity [2,5,7,9,11], while simple drainage should not be performed due to the high associated mortality and recurrence rate [9]. There is correlation between recurrence of the cyst and retroperitoneal location, because they are technically more difficult to excise [5].

## 4 Conclusion

Although mesenteric cysts are a rare condition, it is important the knowledge of these tumors, especially in cases where an intraabdominal mass is felt on the physical examination, so the properly treatment can be performed. If physical examination and imaging tests fail to provide the correct diagnosis, surgery should be indicated for the purpose of diagnosis and treatment, with laparoscopy being the preferred technique whenever possible.

## Provenance and peer review

Not commissioned, externally peer-reviewed.

## Ethical approval

Since we didn't use study of others medical records besides the patient of the case, it was not necessary to submit the paper to the Ethics Committee of our institution. However, the patient consented to carry out this work and all ethical standards have been respected in this work.

## Sources of funding

The authors declare that did not receive any specific grant from funding agencies in the public, commercial, or not-for-profit sectors.

## Consent

The patient consented to carry out this work and all ethical standards have been respected in this work (Written informed consent was obtained from the patient for publication of this case report and accompanying images. A copy of the written consent is available for review by the Editor-in-Chief of this journal on request).

## Guarantor

Nubyhelia Maria Negreiro de Carvalho - nubyhelia@gmail.com.

## Declaration of competing interest

The authors declare that they have no competing interests and this research did not receive any specific grant from funding agencies in the public, commercial, or not-for-profit sectors.
